# Supplementary material for: Identification and functional analysis of non-coding regulatory small RNA FenSr3 in Bacillus amyloliquefaciens LPB-18
Source: PeerJ. 2023 May 15;11:e15236. doi: 10.7717/peerj.15236 (PMC10194069; doi:10.7717/peerj.15236)
Supplement: Supplemental Information 4 [file peerj-11-15236-s004.zip › KO/CK-vs-T1_map/map05170.html]

KEGG PATHWAY: Human immunodeficiency virus 1 infection - Reference pathway


|  |  |
| --- | --- |
| **Human immunodeficiency virus 1 infection - Reference pathway** |  |

[
Pathway menu
| Organism menu
| Pathway entry
| Show description
| User data mapping
]

Human immunodeficiency virus type 1 (HIV-1) , the causative agent of AIDS (acquired immunodeficiency syndrome), is a lentivirus belonging to the Retroviridae family. The primary cell surface receptor for HIV-1, the CD4 protein, and the co-receptor for HIV-1, either CCR5 or CXCR4, are found on macrophages and T lymphocytes. At the earliest step, sequential binding of virus envelope (Env) glycoprotein gp120 to CD4 and the co-receptor CCR5 or CXCR4 facilitates HIV-1 entry and has the potential to trigger critical signaling that may favor viral replication. At advanced stages of the disease, HIV-1 infection results in dramatic induction of T-cell (CD4+ T and CD8+ T cell) apoptosis both in infected and uninfected bystander T cells, a hallmark of HIV-1 pathogenesis. On the contrary, macrophages are resistant to the cytopathic effect of HIV-1 and produce virus for longer periods of time.

|  |  |  |
| --- | --- | --- |
| Reference pathway | 184% 150% 122% 100% 82% 67% 55% | 图片下载 |
